# Female political representation and the gender health gap: a cross-national analysis of 49 European countries

**DOI:** 10.1093/eurpub/ckac122

**Published:** 2022-09-10

**Authors:** Aaron Reeves, Chris Brown, Johanna Hanefeld

**Affiliations:** Department of Social Policy and Intervention, University of Oxford, Oxford, UK; International Inequalities Institute, London School of Economics and Political Science, London, UK; WHO European Office for Investment for Health and Development, Venice, Italy; Department of Global Health and Development, London School of Hygiene and Tropical Medicine, London, UK; Robert Koch Institute, Berlin, Germany

## Abstract

**Background:**

Does increased female participation in the social and political life of a country improve health? Social participation may improve health because it ensures that the concerns of all people are heard by key decision-makers. More specifically, when women’s social participation increases this may lead to health gains because women are more likely to vote for leaders and lobby for policies that will enhance the health of everyone. This article tries to examine whether female participation is correlated with measures of health inequality.

**Methods:**

We draw on data from the World Health Organization Health Equity Status Report initiative and the Varieties of Democracy project to assess whether health is better and health inequalities are smaller in countries where female political representation is greater.

**Results:**

We find consistent evidence that greater female political representation is associated with lower geographical inequalities in infant mortality, smaller inequalities in self-reported health (for both women and men) and fewer disability-adjusted life-years lost for women and men. Finally, we find that greater female political representation is not only correlated with better health for men and women but is also correlated with a smaller gap between men and women because men seem to experience better health in such contexts.

**Conclusions:**

Greater female political representation is associated with better health for everyone and smaller inequalities.

## Introduction

The United Nations Sustainable Development Goals (SDGs) call on countries to ‘achieve gender equality’ (SDG 5). As yet, however, gender equality remains an attainable but unrealized aspiration in all too many parts of the world.^1^^,^^2^ Women often remain excluded from politics, the labour market and other aspects of social life.^3^ Few countries in the world have ever had a situation in which more women than men hold positions in their most important political institutions, and women are still frequently paid less than men for the same work.^4^^,^^5^ Beyond politics, economic and social elites across Europe continue to be dominated by men.^6^^,^^7^ Despite important gains, progress towards gender equality has been ‘uneven’, and in some countries, it has ‘stalled’ entirely.^5^

Fostering gender equality is normatively desirable and may also enhance human flourishing and reduce inequalities.^8^ Gender equality often entails greater female participation in the social and political life of communities, and participation informs decision-making. Those who have power are under no obligation to heed the viewpoints of those who do not or cannot make their voices heard, even in democracies, so the perspectives of those without power are often marginalized.^9^ When people can make their voices heard through formal and informal channels (such as by voting, writing letters, striking, donating or lobbying), decision-makers listen.^10^^,^^11^ Formally allowing previously excluded groups to hold positions of power over resources ensures that the views of marginalized groups are more likely to be taken seriously.^12^ This has important implications for health, as excluding women from full ‘social participation’ in the political life of their communities means they are not able to be involved in, and influence, decisions affecting their health.^13^ Given this, we might expect, therefore, that female political participation would be associated with better health for women.

Women’s social participation could also improve the health other groups in society. This is because the particular location of women in society generates, on average, dispositions and preferences that appear to be different from men.^8^ As a result, when women are given influence over decision-making in the political system they are more likely to push forward changes that accelerate economic development and improve child health.^12^^,^^14^^,^^15^ For example, the extension of female suffrage in US states led to rapid changes in local public health spending and other campaigns that reduced infant mortality.^16^ Greater gender equality in the political system may also reduce inequalities in health by ensuring greater investment in infrastructure such as universal health coverage and cleaner air and water, bringing benefits to the whole community.^12^ When women are excluded from participating in other forms of power-brokering institutions, such as unions, both men and women lose out.^17^ Female participation therefore may improve health for men and women while minimizing inequalities between advantaged and disadvantaged groups.

Despite gender equality and the equal rights of women being enshrined in law, including in the Universal Declaration of Human Rights, political institutions and other governance mechanisms can still sometimes exclude women from participating.^18^ Political institutions and their histories shape who has the right to speak and the conditions under which their voices are heard, and women have often been excluded from these sites of power, both formally and informally.^19^ Policy decisions may influence whether communities and individuals are capable of participating in the political process through, for instance, voter suppression laws or redistricting, which ensure some voices become marginalized.^20^^,^^21^ Political exclusions can persist informally even when the rules excluding them have been removed.^22^

Even when women can vote or hold political office, social participation goes beyond mere involvement: it is not just a type of communication or a form of consultation.^13^ Genuine social participation is more than simply being given the right to vote, for example, and instead requires the capacity to influence the outcome of decision-making processes.^13^

In this article, we go beyond earlier country-specific studies to examine the association between greater female participation in politics and (i) overall health outcomes and (ii) health inequality. We have some evidence that gender equality is correlated with better health for women and men^23–26^ and that there are considerable gender inequalities in political participation.^4^^,^^27^ We also seek to contribute to debates on whether political institutions affect health outcomes and health inequalities. Emerging evidence suggests the nature of political engagement—whether, for instance, processes are participatory and if they are accountable—are correlated with longevity,^28^^,^^29^ but we know far less about whether making these institutions more inclusive is also associated with health inequality. We focus, therefore, on the synergies between the effects of gender equality (SDG 5) in political participation and their role in health outcomes (SDG 3), specifically the consequences on health equity (SDG 10).

## Methods

Numerous measures of health performance were collated for the World Health Organization (WHO) Regional Office for European as part of the Health Equity Status Report initiative (HESRi).^30^ We focus on two sets of variables. The first set are those traditionally associated with good population health (life expectancy at birth and infant mortality per 1000 live births). For infant mortality, we also examine inequalities between richer and poorer regions within countries, measured at the NUTS 2 level within countries. The second set are measures related to environmental health, such as disability-adjusted life-years (DALYs) lost due to inadequate sanitation and air pollution, because there is some evidence that female political leaders invest more in public goods (like clean water) that benefit everybody.^12^ Crucially, we also examine health inequalities within men and women. The inequalities by income (measured using household surveys) are the absolute differences between the poorest and the richest income quintiles. This diverse range of indicators capture different aspects of health and wellbeing, including early years (infant mortality), working life (self-reported health and DALYs) and mortality (life expectancy). Moreover, they make use of the best sources available on health inequality across the set of countries that are the focus of this study. Data have been collected for around 49 countries in the WHO European Region, which comprises 53 Member States and reaches well beyond the European Union. This health inequalities dataset is the most comprehensive ever produced for the WHO European Region. Data cover 1991–2017 and descriptive statistics are reported in Supplementary table S1 (in Supplementary table S2, we include the country-specific observations for each analysis).

We take these measures of health and health inequalities and combine them with measures of political systems created by the Varieties of Democracy project.^31^ V-Dem, as it is commonly known, represents the most systematic and comprehensive measure of the degree and type of democracy across countries over time. We focus on measures that have relevance to female political representation,^32^ such as the measure of the degree of gender equality in politics, a composite indicator that is higher when: (i) countries have instituted fundamental civil liberties for women; (ii) women can and do participate in civil society organizations (CSOs); and (iii) women hold formal political positions, such as a seat in a national parliament. Here, we are particularly interested in capturing a range of forms of social participation. These data were collected through survey questions directed to country experts.^31^ We also collected data on the proportion formal parliamentary seats held by women from the Quality of Government dataset.^33^

We use fixed-effects linear-regression models for the following outcomes: infant mortality, inequalities in infant mortality, inequalities in self-rated health, DALYs due to air and sanitation and life expectancy. For these measures, we have repeated observations within countries over time, as these models allow us to account for country-specific differences that are relatively stable. The models enable us to understand what happens to health, on average, when the political representation of women improves, providing more confidence that the associations we report are not simply due to confounding. However, for a few outcomes (infant mortality and DALYs due to air and sanitation), we also use multilevel regression models with random intercepts to analyse these data, allowing us to visualize the associations and to explore robustness.

All analyses account for gross domestic product (GDP) per capita, because economic development is associated with both gender equality in politics and health outcomes. In our multilevel models, we also adjust for whether a country has ever been a communist country or not, as post-communist countries typically have fewer female politicians and poorer health outcomes.^34^ We have not included an extensive list of control variables because of the relatively small number of observations in the dataset. Standard errors are clustered at the country level and all analyses were conducted in Stata 15.

## Results

We start by examining the correlation between gender equality and infant mortality (Supplementary table S3). When looking at the data cross-sectionally, countries with greater female political participation have lower infant mortality than those with more gender inequality. Even when we look at change over time (using fixed-effects models), we find that a 10-point increase in the political participation of women is associated with ∼2 fewer infant deaths per 1000 live births [*β* = −2.38, 95% confidence interval (CI): −1.14 to −3.61].

We also look at inequalities in infant mortality between more and less affluent parts of each country. Here, too, we find that, adjusting for time and changes in GDP per capita, inequalities in infant mortality within a country are smaller in places where gender equality has gone up than in places where it has not (*β* = −11.40, 95% CI: −4.24 to −18.56).

**Figure 1:**
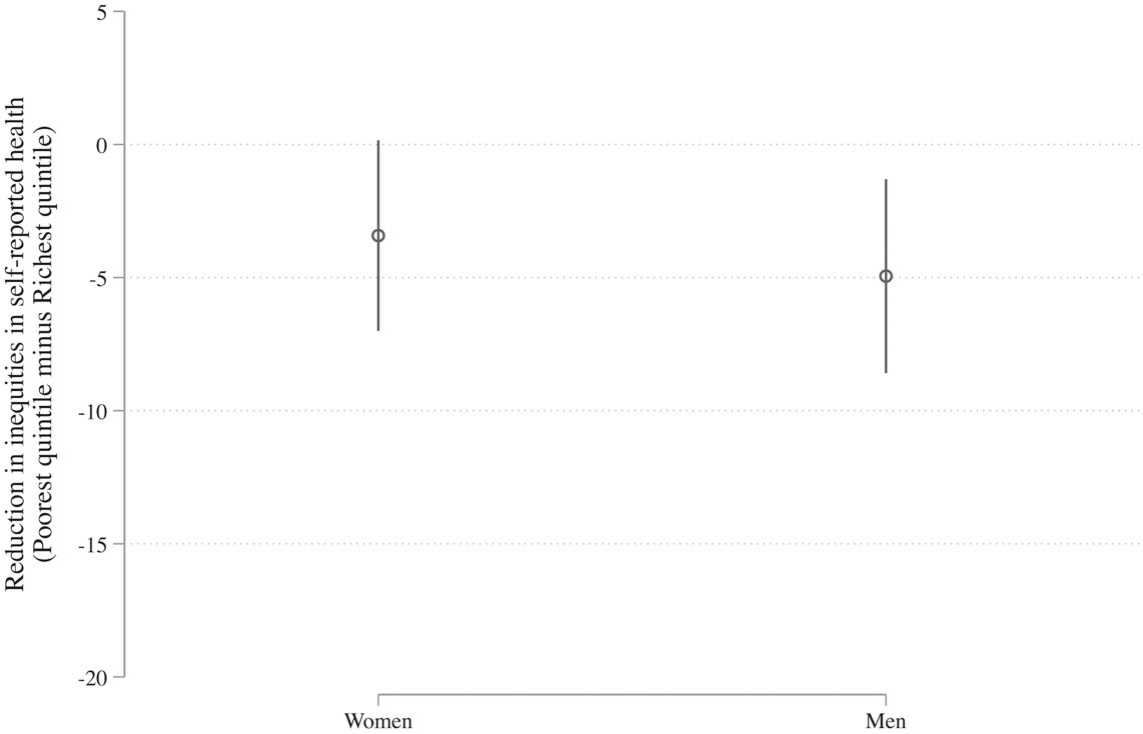
Increased gender equality is associated with reduced inequalities in self-reported health between rich and poor for both men and women

Beyond children, is greater female participation in the political process also correlated with the health and well-being of adults? Here, again, we use repeated observations from within countries over time (using fixed-effects models) to test whether the size of these inequalities in well-being vary according to the level of female political participation. We find a negative correlation (figure 1). When gender equality increases within a country, even though the confidence intervals just cross zero (*β* = −3.42, 95% CI: 0.28 to −7.13, *n* = 418), the gaps in self-reported health between richer and poorer women generally seem to decline. This reduction in the gap is due to better health among the poorest women. Women are not the exception, however: we also see the same association among men (*β* = −4.95, 95% CI: −1.18 to −8.72, *n* = 420). The similarities between these two estimates reinforce each other, suggesting that the difference in self-reported health for richer and poorer men is smaller in countries with more female empowerment.

Our data also indicate that men experience a higher disease burden (represented by DALYs) resulting from unsafe sanitation and air pollution. Analysis of the HESRi Health Equity Dataset showed that men lost 1715.22 DALYs (age-standardized rate per 100 000 population) owing to air pollution while women lost only 894.45 DALYs (age-standardized rate per 100 000 population). The gender gap was far smaller for DALYs lost due to unsafe sanitation, but men still had a higher disease burden. The size of the gender gap also varied considerably across countries: it was very wide in some countries and almost zero in others.

**Figure 2:**
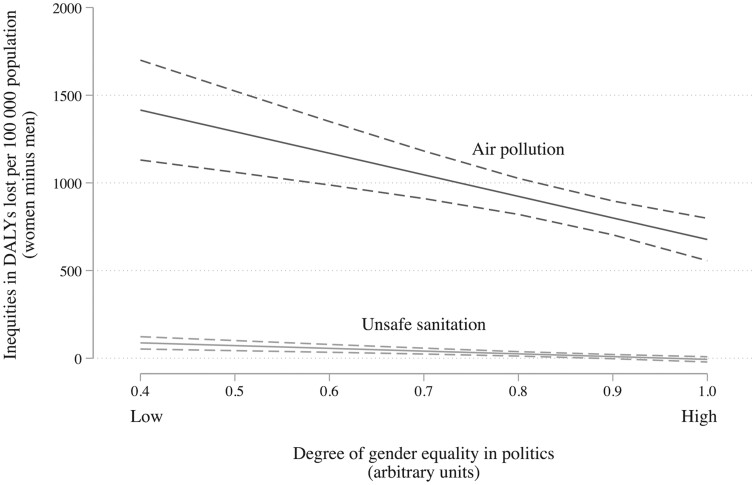
Difference in the number of DALYs lost to unsafe sanitation and air pollution between men and women across 48 countries of the WHO European Region *Note*: Solid line: regression estimate of the difference in DALYs lost between men and women; dashed lines: 95% CI. The source for the degree of gender equality in politics was the Varieties of Democracy project dataset, in which data were collected through survey questions directed to country experts.^32^

Crucially, cross-country variation in the size of the gender gap in DALYs lost through air pollution and unsafe sanitation is correlated with the degree of female political representation.^31^ In countries where women had more involvement in parliament and CSOs, the level of gender inequity in DALYs caused by unsafe sanitation and air pollution was lower. When we look at change over time, we see that the gender inequality in DALYs lost due to air pollution falls (*β* = −175.98, 95% CI: −91.24 to −260.71, *n* = 190) when women become more involved in the political process, even after adjusting for time and changes in GDP per capita. We also find a negative correlation for DALYs lost to sanitation, but this is less certain (*β* = −8.51, 95% CI: −32.90–15.89, *n* = 190), in part because there is far less inequality in DALYs lost to sanitation between men and women (figure 2). These reductions in inequality occur not because women are worse off in countries with more equal political systems. Rather, our data suggest reduced inequality occurs because men’s health improves—the differences have levelled up.

Finally, we consider our most general health outcome, life expectancy at birth. This aggregate measure captures all of the outcomes we have analysed so far, but also incorporates other unmeasured health outcomes. Women usually live longer than men but, again, the size of the gap varies across countries. Life expectancy is also correlated with the degree of political equality between men and women: when women’s political representation within a country increases (using country fixed-effects) we find life expectancy for both men and women is higher in the years following (table 1). Crucially, greater female political participation does not exacerbate the inequalities that already exist between men and women; if anything, there seems to be a slight narrowing of the gap between the groups, although this is not different from zero using conventional thresholds (*P *=* *0.24).

**Table 1:** Greater gender equality is associated with higher life expectancy for both men and women

	Female life expectancy at birth (logged)	Male life expectancy at birth (logged)	Difference between male and female life expectancy at birth
Covariates	(1)	(2)	(3)
10-unit increase in the degree of gender equality	0.012^**^	0.015^**^	−0.0030
	(0.0031)	(0.0051)	(0.0025)
$100 increase in GDP per capita, PPP (constant 2011 international $)	0.00017^**^	0.00027^**^	−0.000098^**^
	(0.000030)	(0.000048)	(0.000021)
Country fixed-effects	Y	Y	Y
Constant	4.20^**^	4.06^**^	0.15^**^
	(0.026)	(0.043)	(0.021)
Observations	1111	1111	1111

PPP, purchasing power parity.

*Notes*: Standard errors in parentheses and are clustered at the country level. Model also adjusts for time and country fixed-effects.

*P <* *0.05,

**P <* *0.01.

**Figure 3:**
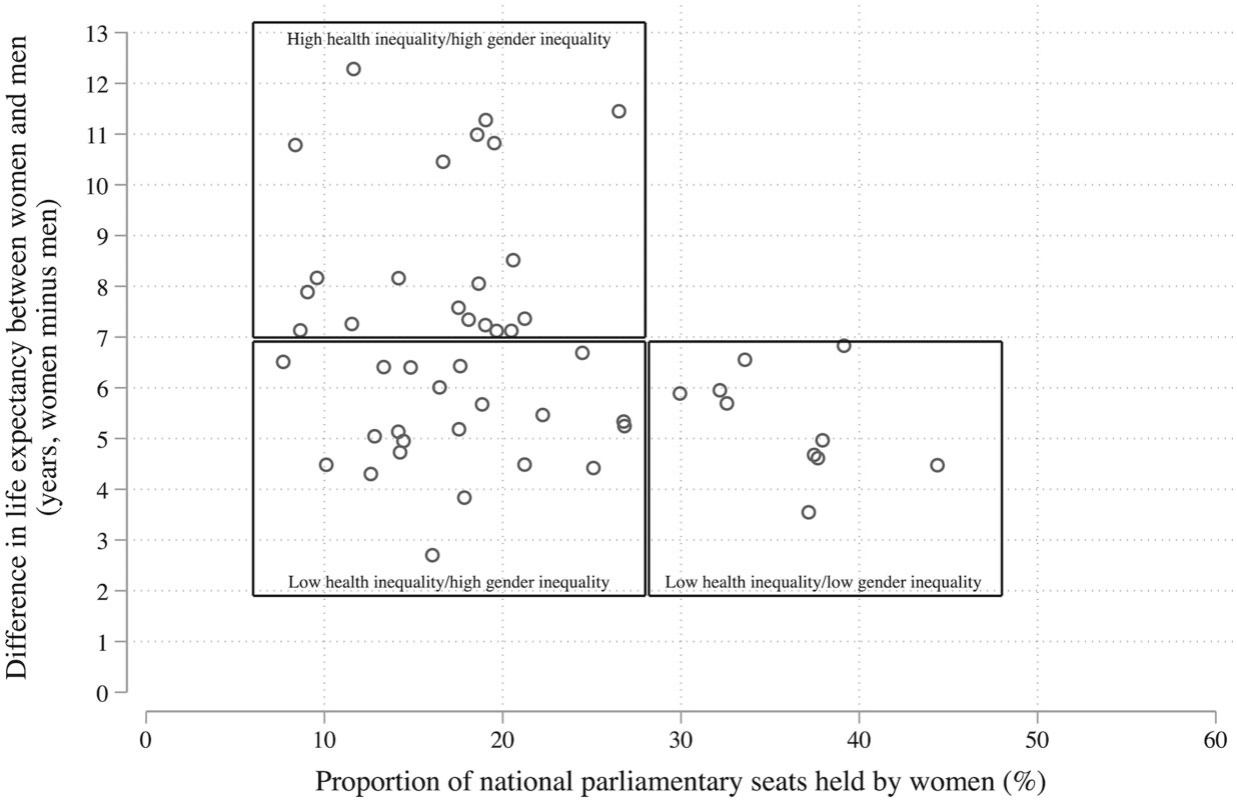
Difference in life expectancy between women and men against the proportion of parliamentary seats held by women across 51 countries of the WHO European Region *Note*: The source for the proportion of national parliamentary seats held by women was the Quality of Government dataset.^34^

As a sensitivity test, we also examine whether these results are consistent with another measure of greater female political participation taken from a different dataset. Here, we focus on the proportion of women in national parliaments from the Quality of Government data.^34^ In a cross-sectional analysis, we find that the life-expectancy gap between women and men is smaller in countries with more women holding formal political positions [i.e. with a parliamentary seat (figure 3)].^33^ This is obviously not a deterministic relationship, but it is striking that no countries with a large proportion of women in parliament had a high level of gender inequity in health. We then model this relationship more formally by exploring whether, after accounting for GDP per capita and time, increases in the proportion of women in parliament are associated with female life expectancy, male life expectancy and the difference between them (see Supplementary table S4). Once again, we find that life expectancy for both men and women is higher in countries that have more female politicians, with some suggestion that women’s health does not benefit more than men’s. In other words, our evidence is consistent with the view that gender equality benefits women, but that it also benefits men.

We also explore whether controlling for changes in the degree of democracy within a country removes the association between women’s empowerment and health. We find that greater women’s empowerment is associated with lower infant mortality and smaller income inequalities in self-reported health in countries irrespective of the degree of deliberative democracy is higher (see Supplementary figure S5).

## Conclusion

We analysed data on health inequity across 49 countries of the WHO European Region alongside measures of gender equality in the political realm. Three key conclusions emerge from our results.

First, countries with more female political representation have lower infant mortality and better life expectancy, and this association holds even after controlling for the level of economic development in each country. This is consistent with earlier work which has documented that women’s empowerment can improve infant and child mortality.^8^^,^^12^^,^^35^ Second, greater gender equality is also correlated with lower socioeconomic inequalities, both between people with different levels of education and between rich and poor parts of the country. Third, gender equality is correlated with better health for men and women. While women tend to have better health than men in our data, the gaps between them in, for example, life expectancy are (depending on the measure) slightly smaller in countries with greater political equality. Importantly, male life expectancy may improve slightly faster than female life expectancy when women have more political power. These findings are consistent with earlier evidence that female political leaders appear to have slightly different political preferences, emphasizing more investment in public health infrastructure that benefits everyone, including men.^12^

Our results add to a growing body of literature that reveals the health benefits associated with gender equality.^36^ Earlier work suggests that the correlation between unemployment and suicide is weaker in countries with more egalitarian gender norms.^23^ Adolescent boys and girls express more life satisfaction when they live in countries with more gender equality.^37^ Countries with more gender equality are twice as likely to report high levels of well-being, half as likely to report being depressed, and about 40% less likely to experience violent deaths.^38^ More specifically, our work unpacks the political roots of health, illuminating how gender equity in the political realm contributes to better health. Here again, our results reinforce earlier studies which found that gender equality is associated with lower infant mortality rates and that exposure to ‘structural sexism’ can increase the likelihood of experiencing chronic conditions and worse physical functioning.^26^^,^^36^

An important implication of our findings is the possibility for policy coherence between SDG 3 (ensuring healthy lives for all), SDG 5 (achieve gender equality and empower all women and girls), SDG 10 (reduce inequality within countries) and SDG 16 (build effective, accountable and inclusive institutions at all levels).^27^ Creating inclusive institutions (SDG 16) that give women power and influence in CSOs and in formal political bodies, such as holding a seat in a national parliament, could, if our data are causal, also accelerate progress on these other goals. While there is some evidence that voting inequality affects health inequalities,^39^ ensuring female participation beyond the ballot box seems particularly important, as a country’s progress on health goals does not always appear to affect the political preferences of voters.^40^ Female political participation in CSOs and formal political institutions may be central to accelerating progress towards achieving health equity.

There are, of course, important limitations to our analysis. Most of the analyses we conduct in this article examine change over time within countries (holding constant those durable differences between countries). The results from these analyses are likely to be closer to the average causal effect than cross-sectional models but it is still possible that time-varying confounders could explain these results. For example, while we do explore the possible confounding role of deliberative democracy for our results (see Supplementary figure S2), it is difficult to separate the degree of democracy from women’s involvement in the political system because these two things have typically moved together. The set of countries we have included in these analyses are also broader than many other similar analyses but this also has the unfortunate side-effect of reducing the number of available country-level covariates that we can include in the model. We have adjusted for GDP per capita, which is likely to be one of the major confounding factors for the associations reported here, but it is entirely possible that other variables could explain our results. Beyond confounding variables, we also cannot see the precise mechanisms through which greater gender equality improves health for all. Important work is already underway in this area, but we will look to future papers to explore why precisely we see the particular relationships documented here. Finally, our analysis has focused on the WHO European Region and the associations may not be generalizable to other parts of the world.

Political equality interacts with other gender norms, and there is a risk that tokenistic inclusion into positions of power without wider shifts in gender relations may not produce clear improvements in health. There nevertheless is some evidence that increasing exposure to female leaders and female labour-force participation changes attitudes to female leadership.^22^

Much of the evidence on health equity demonstrates that a just society is better for the health of everyone and our analyses reinforce this broader point.^36^ Gender equality (one feature of a just society) is associated with better health for women, men, and children. Our analysis, then, provides new insights into the forms of social justice that may play an important role in reducing health inequalities. It adds to the growing body of evidence that demonstrates the importance of the political process for achieving better health for all. Creating inclusive political spaces that facilitate genuine participation from women may improve health and reduce health equity for all.

## Replication

Data and code to replicate these analyses will be posted on Github: https://github.com/asreeves/politics-gender-health.

## Supplementary data

Supplementary data are available at *EURPUB* online.

## Funding

This work has been partially funded by the World Health Organization-EURO (J.H. and A.R.) and the Wellcome Trust (220206/Z/20/Z to A.R.).

*Conflicts of interest*: Both A.R. and J.H. received funding from the World Health Organization for time spent on this research. A.R. had full access to the underlying data and the funder had no influence on the decision to publish. The results do not necessarily represent the views of the WHO European Region.

Key points

- Greater female political representation is associated with lower geographical inequalities in infant mortality.
- Greater female political representation is associated with both better health for men and women and smaller inequalities between men and women.
- Female political representation is good for everyone’s health, including men’s.

## Supplementary Material

ckac122_Supplementary_Data
