# Supplementary material for: Female political representation and the gender health gap: a cross-national analysis of 49 European countries
Source: Eur J Public Health. 2022 Sep 10;32(5):684–9. doi: 10.1093/eurpub/ckac122 (PMC9527963; doi:10.1093/eurpub/ckac122)
Supplement: ckac122_Supplementary_Data [file ckac122_supplementary_data.docx]

Table S1. Descriptive statistics for main variables

| Variable | Obs | Mean | Std. dev | Min | Max | Source |
| --- | --- | --- | --- | --- | --- | --- |
| Infant mortality, 1000 live births | 1,150 | 10.28 | 12.80 | 1.80 | 88.70 | World Bank |
| Absolute inequality (between richest and lowest regions) in infant mortality | 57 | 11.61 | 12.11 | -3.64 | 42.09 | World Bank and Regional Human Development Indicators |
| Absolute inequality (by income) in proportion of women reporting good health | 431 | 16.59 | 5.16 | -7.59 | 28.89 | European Social Survey and EU-SILC |
| Absolute inequality (by income) in proportion of men reporting good health | 433 | 16.75 | 5.27 | -6.92 | 39.29 | European Social Survey and EU-SILC |
| Absolute difference between men and women in DALYs due to air polution | 200 | 820.77 | 598.59 | 65.15 | 2534.46 | Global Burden of Disease Study 2016 |
| Absolute difference between men and women in DALYs due to poor sanittaion | 200 | 13.80 | 53.96 | -87.33 | 375.48 | Global Burden of Disease Study 2016 |
| Life expectancy at birth, women (logged) | 1,138 | 4.36 | 0.05 | 4.20 | 4.46 | World Bank |
| Life expectancy at birth, men (logged) | 1,138 | 4.27 | 0.08 | 4.05 | 4.43 | World Bank |
| GDP per capita, PPP (constant 2011 international $) | 1,205 | 25250.70 | 17439.85 | 1046.78 | 97864.20 | World Bank |
| Women political empowerment index | 1,162 | 8.59 | 1.28 | 3.47 | 9.76 | Varieties of Democracy |
| Proportion of parliamentary seats held by women | 1,018 | 21.03 | 10.59 | 0.00 | 50.00 | Quality of Government |

Table S2. Number of country-specific observations in regressions models for each specific outcome.

| **Country** | **Infant Mortality** | **Infant Mortality Inequality** | **Self-rated health Women by Income** | **Self-rated health Men by Income** | **DALYS due to air pollution** | **DALYS due to sanitation** | **Life expectancy (women)** | **Life expectancy (women)** |
| --- | --- | --- | --- | --- | --- | --- | --- | --- |
| Albania | 19 | 1 |  |  | 4 | 4 | 18 | 18 |
| Armenia | 26 | 4 |  |  | 4 | 4 | 25 | 25 |
| Austria | 27 |  | 14 | 14 | 4 | 4 | 26 | 26 |
| Azerbaijan | 23 | 1 |  |  | 4 | 4 | 22 | 22 |
| Belarus | 23 | 1 |  |  | 4 | 4 | 22 | 22 |
| Belgium | 27 |  | 14 | 14 | 4 | 4 | 26 | 26 |
| Bosnia and Herzegovina | 18 |  |  |  | 4 | 4 | 17 | 17 |
| Bulgaria | 27 |  | 13 | 13 | 4 | 4 | 26 | 26 |
| Croatia | 23 |  | 8 | 8 | 4 | 4 | 22 | 22 |
| Cyprus | 17 |  | 12 | 12 | 4 | 4 | 17 | 17 |
| Czech Republic | 25 |  | 13 | 13 | 4 | 4 | 24 | 24 |
| Denmark | 27 | 1 | 14 | 14 | 4 | 4 | 26 | 26 |
| Estonia | 25 |  | 14 | 14 | 4 | 4 | 24 | 24 |
| Finland | 27 |  | 14 | 14 | 4 | 4 | 26 | 26 |
| France | 27 |  | 13 | 13 | 4 | 4 | 26 | 26 |
| Georgia | 19 | 1 |  |  | 4 | 4 | 18 | 18 |
| Germany | 27 |  | 12 | 12 | 4 | 4 | 26 | 26 |
| Greece | 27 |  | 14 | 14 | 4 | 4 | 26 | 26 |
| Hungary | 26 |  | 13 | 13 | 4 | 4 | 25 | 25 |
| Iceland | 17 |  | 12 | 13 | 4 | 4 | 17 | 17 |
| Ireland | 27 |  | 13 | 13 | 4 | 4 | 26 | 26 |
| Israel | 17 | 12 | 4 | 5 | 4 | 4 | 17 | 17 |
| Italy | 27 |  | 13 | 13 | 4 | 4 | 26 | 26 |
| Kazakhstan | 25 | 6 |  |  | 4 | 4 | 24 | 24 |
| Kyrgyzstan |  | 4 |  |  | 4 | 4 | 25 | 25 |
| Latvia | 24 |  | 13 | 13 | 4 | 4 | 23 | 23 |
| Lithuania | 23 |  | 12 | 12 | 4 | 4 | 22 | 22 |
| Luxembourg | 17 |  | 13 | 13 | 4 | 4 | 17 | 17 |
| Macedonia |  | 1 | 7 | 7 | 4 | 4 | 18 | 18 |
| Montenegro | 11 |  | 2 | 2 | 3 | 3 | 11 | 11 |
| Netherlands | 27 |  | 13 | 13 | 4 | 4 | 26 | 26 |
| Norway | 27 |  | 14 | 14 | 4 | 4 | 26 | 26 |
| Poland | 27 |  | 12 | 12 | 4 | 4 | 26 | 26 |
| Portugal | 27 |  | 14 | 14 | 4 | 4 | 26 | 26 |
| Republic of Moldova |  | 1 |  |  | 4 | 4 | 18 | 18 |
| Romania | 27 |  | 13 | 13 | 4 | 4 | 26 | 26 |
| Russian Federation | 26 | 11 | 4 | 4 | 4 | 4 | 25 | 25 |
| Serbia | 12 |  | 4 | 4 | 4 | 4 | 11 | 11 |
| Slovakia |  |  | 12 | 12 | 4 | 4 | 18 | 18 |
| Slovenia | 27 |  | 13 | 13 | 4 | 4 | 26 | 26 |
| Spain | 27 |  | 14 | 14 | 4 | 4 | 26 | 26 |
| Sweden | 27 |  | 13 | 13 | 4 | 4 | 26 | 26 |
| Switzerland | 27 |  | 10 | 10 | 4 | 4 | 26 | 26 |
| Tajikistan | 26 | 2 |  |  | 4 | 4 | 25 | 25 |
| Turkey | 27 | 3 | 10 | 10 |  |  | 26 | 26 |
| Turkmenistan | 18 | 3 |  |  | 4 | 4 | 17 | 17 |
| Ukraine | 26 | 3 | 3 | 3 | 4 | 4 | 25 | 25 |
| United Kingdom | 27 |  | 12 | 12 | 4 | 4 | 26 | 26 |
| Uzbekistan |  | 2 |  |  | 4 | 4 | 18 | 18 |

Table S3. Greater gender equality is associated with higher infant mortality

|  | Infant mortality  (logged) | Infant mortality  (logged) | Inequalities in infant mortality |
| --- | --- | --- | --- |
| Covariates | (1) | (2) | (3) |
| 10-unit increase in the degree of gender equality | -7.97^**^ (0.41) | -8.12^**^ (3.01) | -11.4^**^ (3.38) |
|  |  |  |  |
| $100 increase in GDP per capita, PPP (constant 2011 international $) | -0.00013^**^ (0.000034) | -0.00016 (0.00017) | -0.00067 (0.00054) |
|  |  |  |  |
| Country fixed-effects | N | Y | Y |
|  |  |  |  |
| Constant | 4.20^**^ (0.026) | 4.06^**^ (0.043) | 0.15^**^ (0.021) |
| Observations | 1053 | 1053 | 57 |

PPP: purchasing power parity.

*Notes*: standard errors in parentheses and are clustered at the country level. Model also adjusts for time and country fixed-effects.

^*^ *p* < 0.05, ^**^ *p* < 0.01.

Table S4. Increase in the proportion of women in parliament improves life expectancy for both men and women

|  | Female life expectancy at birth  (logged) | Male life expectancy at birth  (logged) | Difference between male and female life expectancy at birth |
| --- | --- | --- | --- |
| Covariates | (1) | (2) | (3) |
| 10-unit increase in the degree of gender equality | 0.00049^**^ (0.00013) | 0.00071^**^ (0.00019) | −0.00022^*^ (0.000092) |
|  |  |  |  |
| $100 increase in GDP per capita, PPP (constant 2011 international $) | 0.00018^**^ (0.000032) | 0.00027^**^ (0.000043) | −0.000090^**^ (0.000019) |
|  |  |  |  |
| Country fixed-effects | Y | Y | Y |
|  |  |  |  |
| Constant | 4.30^**^ (0.0069) | 4.18^**^ (0.0098) | 0.12^**^ (0.0044) |
| Observations | 926 | 926 | 926 |
| *R*^2^ | 0.80 | 0.79 | 0.54 |

PPP: purchasing power parity.

*Notes:* standard errors in parentheses and are clustered at the country level. Model also adjusts for time and country fixed-effects.

^*^ *p* < 0.05, ^**^ *p* < 0.01.

Figure S5: The association between women’s empowerment and health does not vary according to the degree of deliberative democracy

A: Infant mortality

*Notes:* The results from this graph come from a linear (fixed-effects) regression model which controls for GDP, time, country dummies, and the degree of deliberative democracy as measured by the Varieties of Democracy project.

B: Life expectancy

*Notes:* The results from this graph come from a linear (fixed-effects) regression model which controls for GDP, time, country dummies, and the degree of deliberative democracy as measured by the Varieties of Democracy project.
